# Repair of Iron Centers RIC protein contributes to the virulence of *Staphylococcus aureus*

**DOI:** 10.1080/21505594.2017.1389829

**Published:** 2017-12-08

**Authors:** Liliana O. Silva, Lígia S. Nobre, Dalila Mil-Homens, Arsénio Fialho, Lígia M. Saraiva

**Affiliations:** a Instituto de Tecnologia Química e Biológica NOVA, Av. da República Oeiras, Portugal; b Institute for Bioengineering and Biosciences (iBB), Instituto Superior Técnico, Av. Rovisco Pais, 1, Lisboa, Portugal

**Keywords:** di-iron protein, *Galleria mellonella*, innate immunity *Staphylococcus aureus*

## Abstract

RICs are a family of bacterial proteins involved in the repair of iron centers containing proteins damaged by the antimicrobial reactive species liberated by the innate immune system of infected hosts. *Staphylococcus aureus* is a human pathogen with increasing antibiotic resistance that also contains a RIC-like protein. In this work, we show that the survival of *S. aureus* within macrophages decreases upon inactivation of *ric*, and that the viability was restored to levels similar to the wild-type strain by reintroduction of *ric* via in trans complementation. Importantly, in macrophages that do not produce reactive oxygen species, the lower survival of the *ric* mutant was no longer observed. In lung epithelial cells, the intracellular viability of the *S. aureus ric* mutant was also shown to be lower than that of the wild-type. The wax moth larvae *Galleria mellonella* infected with *S. aureus ric* mutant presented an approximately 2.5-times higher survival when compared to the wild-type strain. Moreover, significantly lower bacterial loads were determined in the larvae hemolymph infected with strains not expressing *ric*, and complementation assays confirmed that this behavior was related to RIC. Furthermore, expression of the *S. aureus ric* gene within the larvae increased along the course of infection with a ~20-fold increase after 8 h of infection. Altogether, the data show that RIC is important for the virulence of *S. aureus*.

*Staphylococcus aureus* colonizes the upper respiratory tract and skin of humans, which constitutes a risk factor for the development of invasive diseases such as pneumonia, meningitis and septicaemia, especially in immune-compromised people.^1^ The success of *S. aureus* infections depends on the ability of the pathogen to escape the host's protective mechanisms, allowing invasion and pathogen proliferation. The multitude of evasive mechanisms and the increase in antibiotic resistance have made *S. aureus* a serious human threat. Moreover, methicillin-resistant *S. aureus* strains (MRSA) are no longer restricted to the hospital setting and are widespread in the community.^2^

The innate immune system is the first barrier encountered by the pathogen during host infection. Several studies have reported that internalisation by epithelial cells and phagocytosis by macrophages exposes the microbes to reactive oxygen species (ROS).^3^ Therefore, the defences against oxidative stress play an important role in pathogen survival. The wax moth larva *Galleria mellonella*, that only has innate immunity, is a recognized *in vivo* model for the study of bacterial virulence of several pathogens including *S. aureus*.^4–6^ *G. mellonella* presents advantages over conventional mammalian models, due to the higher temperature required for infection (37°C) and the possibility of the direct injection of a precise inoculum.^7,8^

Repair of Iron Centers (RIC) proteins are a widespread family of bacterial proteins, which are also present in the genomes of *Trichomonas vaginalis* and *Cryptococcus neoformans* eukaryotes.^9^ The first RIC protein was found in *E. coli* due to the marked induction of the encoding gene (formerly named *ytfE)* in cells grown under nitrosative stress conditions.^10^ Consistent with these results, transcriptomic studies done in several organisms have consistently shown that the expression of *ric* is induced in stressed cells, and that the *ric* gene deletion generates strains with lower resistance to nitrosative stress.^11,12^ However, *in vitro* studies done in *S. aureus* indicated that the protein protects from oxidative stress as inactivation of *ric* decreased the viability of *S. aureus* when exposed to hydrogen peroxide.^9^ Furthermore, the *S. aureus* Δ*ric* mutant strain exhibited reduced activity of important iron-sulfur (FeS)-containing enzymes such as aconitase and fumarase, activities that could be recovered to the levels observed in the wild-type strain by addition of the recombinant RIC protein to the cell extracts.^9^ Following these studies, in this work we have analysed the contribution of RIC to the survival of *S. aureus* during infection of macrophages, lung epithelial cells, and *G. mellonella* larvae.

Therefore to investigate the *in vivo* role of *S. aureus* RIC, we first tested the behaviour of a strain lacking *ric* during infection of innate immune cells. For this purpose, macrophages J774A.1 were incubated with *S. aureus* wild-type and an isogenic Δ*ric* mutant, and the survival rate of each strain was determined (Fig. 1). When compared with the parental strain, *S. aureus* Δ*ric* exhibited lower resistance to macrophage killing. Moreover, the susceptibility of the mutant was shown to be dependent on the infection time, *i.e*., during the first half-hour post-infection no major differences were observed in the number of viable colonies between the two strains, whereas after 6 h a decrease of ∼40% in the survival rate was noted for the Δ*ric* mutant strain. Additionally, expression in *trans* of RIC abolished the increased susceptibility of the mutant strain, that under these conditions exhibited a viability similar to that of the wild-type (Fig. 1A).

**Figure 1.**
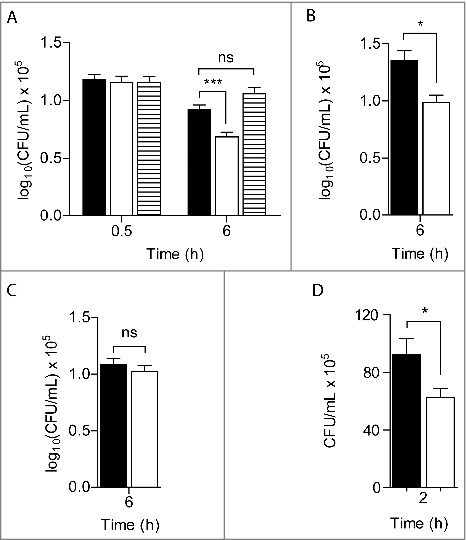
RIC protects *S. aureus* from phagocyte producing ROS. Macrophages J774A.1 were infected, at an MOI ∼ 5, with *S. aureus* JE2 (black bar), JE2 *∆ric* (white bar) and JE2 Δ*ric* carrying the complementation vector (pMK4-RIC– striped bar) following activation by INF-γ/LPS (A), and in the presence of the mammalian iNOS inhibitor L-NMMA (B) or the NADPH phagocyte oxidase inhibitor apocynin (C). Bacterial counts were determined at 0.5 or 6 h post-infection. In (D) is depicted the quantification of intracellular bacteria after infection of lung epithelial A549 cells with *S. aureus* JE2 (black bar) and JE2 Δ*ric* (white bar), for 2 h. Data represent means of three (A, B and C) and two (D) biological samples analysed in triplicate, with standard error and unpaired Student's t-test (***P < 0.0005; *P < 0.05; ns: not significant).

Treatment of macrophages with LPS and γ-IFN induces enzymes such as nitric oxide synthase (iNOS) and the NADPH oxidase (NOX), which produce RNS and ROS that destroy bacteria. To elucidate the role of RIC in the survival of *S. aureus* against these species, infection experiments were also performed in macrophages treated, separately, with inhibitors of iNOS and NOX, namely L-NMMA and apocynin, respectively.

In macrophages treated with L-NMMA, that did not produce NO as attested by nitrite quantification (Figure S1), the viability of the *S. aureus* *ric* mutant was still lower than that of the wild-type strain (Fig. 1B). On the contrary, the survival of the *ric* mutant and of wild-type was similar in macrophages treated with apocynin (Fig. 1C). As apocynin is shown to not interfere with production of nitric oxide (Figure S1), these results indicate that RIC protects *S. aureus* from the oxidative stress imposed by macrophages.

*S. aureus* is a common inhabitant of the human skin and mucosal surfaces that uses internalization into epithelial cells as an immune evasion mechanism.^13^ To further understand the role of RIC in *S. aureus* survival, we carried out invasion/survival assays with human lung epithelial A549 cells, which is the most commonly used epithelial cell model for the study of *S. aureus*.^14^ After 30 min of incubation of the A549 cells with a similar number of wild-type and mutant strain bacterial cells (∼10^7^ CFU/mL), no differences in the intracellular survival between the two strains was observed (1.4 × 10^6^ and 1.2 × 10^6^, respectively). However, after 2 h of infection the intracellular viability of the *S. aureus* *ric* mutant in the epithelial cells was lower than that of the wild-type strain (Fig. 1D). These results show that RIC contributes to the successful infection of non-phagocytic cells, such as the lung epithelial cells, by *S. aureus*.

*Galleria mellonella*, that is a model organism for the study of innate immunity,^15^ was here used to determine the virulence of *S. aureus*. For this purpose, groups of larvae were injected with *S. aureus* JE2, incubated at 37°C, and the survival rate was recorded daily for up to 4 days. While inoculation of PBS exhibited no effect (data not shown), the administration of live MRSA (JE2) strain reduced the larval survival in a dose-dependent manner. Inoculation of 10^3^ CFU/larvae reduced larval viability by 30% after 3 days and 10^8^ CFU/larva caused the death of all larvae after 2 days (Fig. 2A). The lethal dose of *S. aureus* showed to cause the death of 50% (LD_50_) of the *G. mellonella* population at 48 h was found to be ∼10^7^ CFU/larva (Figure S2).

**Figure 2.**
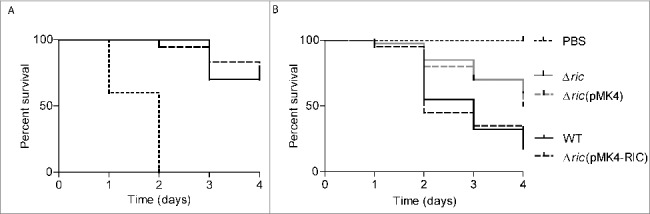
*S. aureus* *ric* mutant has decreased ability to infect *Galleria mellonella*. A. Survival of *G. mellonella* after infection with *S. aureus* JE2 at the following bacterial CFU per larva: 10^3^ (solid line), 10^5^ (dashed line), and 10^8^ (dotted line).B. *G. mellonella* was infected with *S. aureus* JE2 (WT; black line), and the isogenic *ric* mutant strain (Δ*ric*; grey line) (***P < 0.0001). For complementation, *G. mellonella* was also infected by *S. aureus* JE2 Δ*ric* carrying the vector pMK4-RIC (black dashed line), or with the empty vector pMK4 (grey dashed line) (**P < 0.005). In B, approximately 10^7^ bacterial cells were injected per larvae. The survival curves were compared using Mantel-Cox test. Ten larvae were analysed in each condition and larval survival was monitored daily. In all cases, no larval death was observed upon administration of PBS.

The role of RIC in *S. aureus* virulence was examined in *G. mellonella* by inoculating larvae with the *S. aureus* wild-type and the correspondent isogenic *ric* mutant. Inoculation of *G. mellonella* with equivalent doses of *S. aureus* JE2 and Δ*ric* mutant showed that the later had a higher survival rate (Fig. 2B). Four days following infection, only ∼20% of the *S. aureus* JE2 wild-type-infected larvae were still alive, whereas survival of larvae infected with the *S. aureus* *ric* mutant was approximately two 2.5-times higher. Complementation *in trans* with a *ric* plasmid-borne restored the lethality of the mutant strain to levels similar to those induced by the wild-type strain (Fig. 2B).

We also assessed the proliferation of *S. aureus* within *G. mellonella* by determination of the bacterial loads in the larvae hemolymph. *S. aureus* wild-type and Δ*ric* mutant were used at concentrations of ∼1 × 10^7^ CFU/larva, and the hemolymph bacterial load was determined at 1 h, 4 h and 8 h post-infection. The viability of the two strains did not decrease significantly after 4 h of infection, but 8 h post-infection the *ric* mutant exhibited an intracellular viability lower than the wild-type strain (Fig. 3A). Moreover, expression of a RIC plasmid-borne in the *ric* mutant cells led to an increase of the mutant strain viability to levels comparable to the wild-type (Fig. 3A).

**Figure 3.**
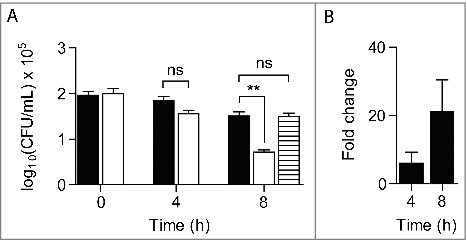
*S. aureus* Δ*ric* mutant has lower intracellular survival in *G. mellonella.* A. Bacterial load was analysed in the larvae hemolymph following incubation with *S. aureus* JE2 (black bar), JE2 Δ*ric* (white bar) and JE2 Δ*ric* carrying pMK4-RIC (striped bar) for 4 h and 8 h. Data represent means of four independent assays analysed with standard error and unpaired Student's t-test (**P < 0.001; ns: not significant). B. Quantitative RT-PCR analysis of the *ric* expression done in total RNA extracted from *G. mellonella* following incubation with *S. aureus* JE2, for 4 h and 8 h, and relative to the initial expression level (time zero of infection). Data was normalized to the expression of the constitutive *16S* rRNA gene. Data represent mean values analysed with standard error (n = 6).

To further investigate the role of *ric* to the *S. aureus* survival during infection of *G. mellonella*, we have determined the expression of the *S. aureus* *ric* gene in the larvae by quantitative RT-PCR analysis. For this purpose, total RNA was extracted from *G. mellonella* after infection with *S. aureus* wild-type up to 8 h. The expression of the *S. aureus* *ric* gene within the larvae increased along the course of infection, been the highest after 8 h of infection (Fig. 3B). These results are consistent with the observed contribution of RIC to the survival of *S. aureus* within the larvae (Fig. 3A).

Previous studies done in *E. coli* and *H. influenzae* have implicated RIC in nitrosative stress resistance on the basis of marked increase of the *ric* gene expression and enhanced sensitivity of the Δ*ric* mutants to nitrosative stress.^10,16^ However, in NO-treated *S. aureus* cells, the *ric* mRNA levels were only slightly increased and the growth of the *S. aureus* Δ*ric* mutant was not significantly compromised.^9,12^ Although, it is not possible to exclude that the presence in *S. aureus* of other RNS defences, particularly flavohaemoglobin^12,17,18^, may mask the role of RIC in NO stress resistance of this microorganism, several results indicate the involvement of RIC in oxidative stress resistance. RIC is herein shown to defend *S. aureus* from the effects caused by oxidative stress imposed by macrophages and consistent with these results, we previously reported that *S. aureus* *ric* mutant is more sensitive to oxidative stress.^9^ Also, the mRNA ric levels increase upon exposure of *S. aureus* to hydrogen peroxide^11^, and a marked up-regulation of the *ric* gene was detected in *Salmonella enterica* and *Yersinia pestis* upon infection of macrophages and rats, respectively.^19,20^ In addition, inactivation of *ric* decreased the survival of *H. influenzae* within macrophages.^16^

*S. aureus* uptake by epithelial cells is a rapid process that is dose-dependent.^21^ We observed that the deletion of ric reduced the rate of *S. aureus* internalization and survival into human epithelial cells. Interestingly, the *S. aureus* *ric* gene is upstream of the *lytSR* gene cluster that encodes a two-component system regulating murein hydrolase activity and cell death.^22^ Moreover, Brunskill and co-workers previously reported that the absence of *ric* in *S. aureus* causes morphological defects^23^, while other authors reported that the *ric* transcription increases upon internalization of *S. aureus* by epithelial A549 cells, along with other genes involved in iron metabolism and oxidative stress resistance.^13^

*Galleria mellonella* wax moth larval infection model has been used to study bacterial pathogenesis, including infections by *S. aureus*.^4–6,24–26^ In particular, *G. mellonella* allowed revealing several *S. aureus* virulence factors, such as the accessory gene regulator (Agr), a global regulator of the staphylococcal virulon that includes secreted virulence factors and surface proteins, RelA, a guanosine pentaphosphate synthase that regulates amino acid biosynthesis under nutrient-limited conditions, and two glyceraldehyde-3-phosphate dehydrogenase homologs called GapAB, involved in glycolysis and gluconeogenesis, respectively.^4–6,27,28^ Our data show that RIC also contributes to the virulence of *S. aureus*.

The inactivation of RIC in *S. aureus* was previously reported to decrease the activity of oxidatively damaged Fe-S containing proteins such as aconitase and fumarase^9^, enzymes that are required for a functional TCA and fully respiratory activity of *S. aureus*. The contribution of RIC to the survival in phagocytes and epithelial cells and to the virulence towards *G. mellonella* shows the *in vivo* importance of this protein, which is most probably related with protection that it confers against the oxidative stress imposed by the host cells.

Experimentally the study included the *S. aureus* strains listed in Table S1, namely the methicillin-resistant *S. aureus* (MRSA) strain JE2 (wild-type), derived from community-associated methicillin-resistant *S. aureus* strain USA300 LAC^29^, and the JE2 Δ*ric* strain, which were provided by the Nebraska Transposon Mutant Library.^30^ The media used for growth of bacteria was purchased from BD Difco and antibiotics from Sigma. *S. aureus* was cultured overnight in Tryptic Soy Broth (TSB), at 37°C, and used to inoculate fresh Luria-Bertani (LB) medium and grown to the indicated optical density at 600 nm (OD_600_). Bacterial viability was determined by CFUs counting by performing serial dilutions of *S. aureus* in phosphate-buffered saline (PBS), plating on Tryptic Soy Agar (TSA) and incubation overnight at 37°C.

The infecting dose was optimized by injecting *G. mellonella* with increasing inocula of *S. aureus* JE2 cell suspensions (∼10^3^–10^8^ cells/larva). For LD_50_ determination, eight bacterial concentrations were tested and the number of dead larvae was evaluated after 48 h, 72 h and 96 h. GraphPad Prism program was used to plot a non-linear fitting curve and obtain the LD_50_ value.

*S. aureus* JE2 and the isogenic Δ*ric* mutant derivative were grown overnight and diluted in PBS (to final 10^7^–10^8^ CFU/mL).

A DNA fragment of ∼1500 bp containing the complete coding region of the *S. aureus* *ric* gene plus a ∼1000 bp upstream region, that contains the *ric* own promoter and lacks any other complete open reading frame, was PCR-amplified from *S. aureus* NCTC8325 genomic DNA using the oligonucleotides SA_RICcomp_fw and SA_RICcomp_rev (Table S2) and ligated into pMK4 digested with *Eco*RI and *Sal*I. After confirming the integrity of the cloned gene, the resulting vector (pMK4-RIC) was electroporated (∼100 ng) into *S. aureus* RN4220 and transformants were selected on TSA medium containing 5 μg/mL chloramphenicol. The recombinant plasmid extracted from *S. aureus* RN4220 cells was electroporated (∼20 *µ*g) into *S. aureus* JE2 Δ*ric*, and its integration confirmed by colony PCR and digestion with appropriated enzymes. Restriction enzymes were obtained from New England Biolabs, DNA polymerase from Roche.

For the eukaryotic cell culture assays, murine macrophages J774.1 and human lung epithelial A549 cells (ATCC CCL 185) were routinely cultivated for 2 days in Dulbecco's modified Eagle's medium (DMEM, Invitrogen) supplemented with 10% fetal bovine serum (DMEMi), 50 U/mL penicillin and 50 *μ*g/mL streptomycin (DMEMc), at 37 °C in a 5% CO_2_-air atmosphere. *S. aureus* JE2 and JE2 Δ*ric* mutant cells grown to OD_600_ of ∼0.4 were collected, washed with PBS and diluted in DMEMi to obtain a culture at OD_600_ ∼0.03. Prior to infection, macrophages (∼5 × 10^5^ cells/mL) were seeded in 24-well plates and cultivated under 5% CO_2_-air atmosphere at 37°C, for 48 h, and activated by incubation with 1 *μ*g/mL gamma interferon (IFN-γ) and 5 *μ*g/mL *E. coli* lipopolysaccharides (LPS), for 5 h; when indicated, 800 *μ*M NG-monomethyl-l-arginine acetate salt (L-NMMA) or 400 *μ*M apocynin were added to inhibit inducible nitric oxide synthase or NADPH phagocyte oxidase, respectively. Bacterial suspensions at ∼10^7^ cells/mL were used to infect macrophages at a multiplicity of infection (MOI) of 5. After a 30 min incubation period, non-phagocytosed bacterial cells were removed by incubation with 50 µg/mL gentamicin for 10 min. Macrophages were washed with PBS, lysed (2% saponin, Sigma), and the intracellular bacterial CFUs determined after 30 min and 6 h of infection. Nitric oxide levels were inferred by the oxidative product nitrite using the Griess assay.^31^

For the internalization assays in lung epithelial cells, 24 h prior to infection, human lung epithelial cells A549 (ATCC CCL 185) (∼2 × 10^5^ cells/mL) were seeded in 24-well plates and cultured in 5% CO_2_-air atmosphere at 37 °C. *S. aureus* wild-type and *ric* mutant strains grown in LB to OD_600_ of ∼0.5–0.6 were diluted in DMEMi to OD600 ∼0.05 (∼10^7^ cells/mL) and incubated for 30 min and 2 h in A549 cells (MOI of 20). Extracellular bacteria were removed by incubation with 4 *μ*g/mL lysostaphin (Sigma) at 37 °C for 20 min. Lung epithelial cells were PBS-washed, trypsinised and lysed with cold Triton X-100 (0.1%), and viable intracellular bacteria were counted.

Larval infection assays were performed with *G. mellonella* larvae reared in an iBB insectarium and maintained on a beeswax and pollen grains diet at 25 °C in darkness, and used in killing assays at the final instar larval stage. The infecting dose was optimized by injecting *G. mellonella* with increasing inocula of *S. aureus* JE2 cell suspensions (∼10^3^–10^8^ cells/larva). *S. aureus* JE2 and the isogenic Δ*ric* mutant were grown overnight and diluted in PBS (to final 10^7^–10^8^ CFU/mL). For each bacterial dilution, 3.5 *μ*L aliquots were injected into the hindmost left proleg of each larva using a microsyringe adapted to a micrometer that controls the volume of injection. Control larvae injected with equal volumes of PBS were also monitored. Larvae were placed in Petri dishes and stored in the dark at 37 °C up to 4 days. Larval survival was monitored daily by inspection for dead organisms which were identified by development of a black colour resultant from larval melanisation, and immobility.

Bacterial load in the *G. mellonella* hemolymph was evaluated in three living larvae that were punctured in the abdomen with a sterile needle and after 1 h (time zero of infection), 4 h and 8 h of infection the plasma was collected to a sterile microtube containing a few crystals of phenylthiourea. The hemolymph was serially diluted in PBS, plated on TSA plates and CFU were determined after incubation at 37 °C, for ∼14 h.

Statistical analysis was carried out using GraphPad Prism version 5.01 for Windows. Survival curves were plotted using the Kaplan-Meier method, and differences in survival were calculated using the Mantel-Cox test for curve comparisons. Ten larvae were examined for each condition, and each experiment was repeated at least three times, over two different weeks and for up to four weeks. Differences between mean values were tested for significance by performing unpaired two-tailed Student's t-test with *P*<0.05.

The *S. aureus* *ric* expression during *G. mellonella* infection was analysed by incubating the larvae with *S. aureus* (∼10^7^ CFU/larva). For each time point (0 h, 4 h and 8 h after infection), three living larvae were cryopreserved, sliced and homogenized in 1 mL TRIzol (ThermoFisher Scientific). Total animal/bacteria RNA was extracted according the manufacturer's protocol, treated with RNase-Free DNase (Quiagen), and its concentration and purity was evaluated in a Nanodrop ND-1000 UV–visible spectrophotometer (Thermo Fisher Scientific) and agarose gel. Total RNA (900 ng) was reverse transcribed with the Transcriptor High Fidelity cDNA Synthesis Kit (Roche) using the Anchored-oligo (dT)18 and Random Hexamer primers. Quantitative real-time RT-PCR assays were done in a LightCycler® 480 (Roche) using the oligonucleotides listed in Table S2 and the LightCycler® 480 SYBR Green I Master kit (Roche). Relative quantification of *ric* gene is shown in relation to the *16S* rRNA reference gene, whose expression does not vary under the tested conditions, and using the comparative CT method.

## Supplementary Material

KVIR_S_1389829.docx
