# Supplementary material for: Repair of Iron Centers RIC protein contributes to the virulence of Staphylococcus aureus
Source: Virulence. 2017 Dec 8;9(1):312–7. doi: 10.1080/21505594.2017.1389829 (PMC5955197; doi:10.1080/21505594.2017.1389829)
Supplement: KVIR_S_1389829.docx [file kvir-09-01-1389829-s001.docx]

**Supplemental Material**

***Staphylococcus aureus* resistance to innate immunity is conferred by the Repair of Iron Centers RIC protein**

Liliana O. Silva^1,+^, Lígia S. Nobre^1,+^, Dalila Mil-Homens^2^, Arsénio Fialho^2^, and Lígia M. Saraiva^1^*

^1^Instituto de Tecnologia Química e Biológica NOVA, Av. da República 2780-157 Oeiras, Portugal

^2^[Institute for Bioengineering and Biosciences](http://ibb.tecnico.ulisboa.pt/) (iBB), Instituto Superior Técnico, Av. Rovisco Pais, 1, 1049-001 Lisboa, Portugal

+ Both authors contributed equally to this work

*Corresponding author:

Lígia M. Saraiva

Av. da República, 2780-157 Oeiras, Portugal

E-mail: lst@itqb.unl.pt

**Table S1.** *S. aureus* strains used in this study

| *S. aureus* strain | Description | Reference |
| --- | --- | --- |
| JE2 | CA-MRSA strain USA300 lac cured of plasmids | NARSA collection |
| JE2 Δ*ric* | JE2 transposon mutant NE1857 (SAUSA300_0253); Δ*ric*::Erm^R^ | NARSA collection |
| JE2 Δ*ric* (pMK4) | JE2 Δ*ric* carrying the *E. coli-S. aureus* shuttle vector pMK4 (Erm^R^; Cm^R^) | This study |
| JE2 Δ*ric* (pMK4-RIC) | JE2 Δ*ric* carrying pMK4 with *S. aureus ric* gene (Erm^R^; Cm^R^) | This study |
| NCTC8325 | MSSA strain cured of prophages | Laboratory stock |
| RN4220 | Restriction-deficient derivative of NCTC8325 | Laboratory stock |

Antibiotic resistance abbreviations: Erm^R^, erythromycin resistance; Cm^R^, chloramphenicol resistance.

**Table S2.** Oligonucleotides used in this study

| **Oligonucleotide** | **Sequence 5’-3’** |
| --- | --- |
| SA_RICcomp_fw | GGTACGGAAGAATTCGAAGTGCG |
| SA_RICcomp_rev | TAGCACGAGTCGACCTCAGTGAA |
| M13_fw | GTAAAACGACGGCCAG |
| M13_rev | CAGGAAACAGCTATGAC |
| SA_RIC_RT_fw | GGGAATAGATTTTTGTTGTGGC |
| SA_RIC_RT_rev | GGTCCATGTACTTTCGATAACTTC |

**Figures**

###

### Figure S1. Nitrite determination in supernatant of infected macrophages.

### Activated murine macrophages J774A.1 in the absence (-) and presence (+) of mammalian iNOS or NADPH oxidase inhibitors (L-NMMA and apocynin, respectively) were infected with S. aureus JE2 (black) or JE2Δric cells (white). Following 6 h of infection, the supernatants were collected, mixed with the Greiss reagent (1:1), and absorbance at 548 nm was measured.

**Figure S2:** **LD_50_ determination of *S. aureus* in *G. mellonella.***

The lethal dose of *S. aureus* leading to 50 % of *G. mellonella* killing was determined using several bacterial concentrations and by counting the number or larvae dead after 48 h. GraphPad Prism program was used to plot a non-linear fitting curve and determine the LD_50_ value.
